# Meetings of Societies

**Published:** 1906-12

**Authors:** 


### MEETINGS OF SOCIETIES.

### Bristol Medico-Chirurgical Society.

*Annual Meeting, October 10th, 1906.*

After a vote of thanks to the retiring President, Mr. John Dacre, had been unanimously passed, Mr. James Taylor took the chair and gave an introductory address (see p. 289). Subsequently cordial thanks were given to Mr. Taylor for his able and interesting address.

The Honorary Secretary, Mr. H. F. Mole, read his annual report. The balance in hand was £147 14s. 6d. on the ordinary account. This was smaller than last year owing to the large grant which had been made to the library.

The Editorial Secretary of the *Journal*, Mr. James Taylor, read his annual report. Mr. C. K. Rudge, the Honorary Librarian of the Medical Library, reported that there were 20,845 volumes in the library, and that 237 current periodicals were regularly received. A complete financial balance sheet was presented. These reports were adopted.

The following officers were chosen for the ensuing year: President-elect—Dr. H. Waldo; Members of Committee—Dr. J. Michell Clarke, Dr. B. M. H. Rogers, Dr. P. Watson Williams, Mr. C. K. Rudge, Dr. James Swain, and Dr. George Parker; and Members of the Library Committee—Mr. C. K. Rudge, Mr. Munro Smith, and Dr. Carey Coombs.

### ACCOUNTS OF THE BRISTOL MEDICO-CHIRURGICAL SOCIETY, Session 1905–06.

| | £ | s. | d. | | £ | s. | d. |
|---|---|---|---|---|---|---|---|
| Carried forward from 1904–05 ... ... ... | 162 | 16 | 2 | Stamps and sundries ... | 2 | 5 | 3 |
| Members' Subscriptions 1905–06 ... ... ... | 166 | 15 | 9 | Grant to Clerk of Library | 5 | 5 | 0 |
| Interest on Deposit Notes | 3 | 11 | 5 | Grant to Library ... ... | 75 | 0 | 0 |
| | | | | Telephone at Medical School ... ... ... ... ... | 3 | 10 | 0 |
| | | | | University College—use of rooms ... ... ... ... ... | 52 | 10 | 0 |
| | | | | Lanterns ... ... ... ... ... | 5 | 9 | 0 |
| | | | | Printing and Stationery... | 3 | 13 | 6 |
| | | | | Porter at School ... ... | 1 | 6 | 1 |
| | | | | To Secretary of *Journal* ... | 36 | 10 | 0 |
| | | | | | £185 | 8 | 10 |
| | | | | Balance in hand ... | 147 | 14 | 6 |
| | £333 | 3 | 4 | | £333 | 3 | 4 |

### "JOURNAL" ACCOUNT, TO OCTOBER, 1906.

| | £ | s. | d. | | £ | s. | d. |
|---|---|---|---|---|---|---|---|
| Carried forward from 1904-05 ... ... ... ... | 11 | 14 | 10 | Balance in hand ... | 13 | 4 | 10 |
| From Editorial Secretary | 1 | 4 | 6 | | | | |
| Interest on Deposit Note | 0 | 5 | 6 | | | | |
| | £13 | 4 | 10 | | £13 | 4 | 10 |

### BRISTOL MEDICAL LIBRARY.

(MEDICO-CHIRURGICAL SOCIETY SECTION.)

*Statement of Income and Expenditure for fifteen months (Oct., 1904, to Dec., 1905).*

| INCOME. | £ | s. | d. | EXPENDITURE. | £ | s. | d. |
|---|---|---|---|---|---|---|---|
| Cash per Hon. Secretary | 110 | 0 | 0 | Fire Insurance (two years) | 2 | 8 | 0 |
| Sale of Duplicate Books | 1 | 3 | 6 | Books ... ... ... ... | 1 | 13 | 0 |
| | | | | Subscriptions ... ... ... | 11 | 7 | 4 |
| | | | | Binding ... ... ... ... ... | 24 | 1 | 8 |
| | | | | Carriage ... ... ... ... ... | 0 | 6 | 3 |
| | | | | Postages and Sundries ... | 1 | 2 | 1 |
| | | | | | £40 | 18 | 4 |
| | | | | Deficit from 1903-04 | 48 | 8 | 3 |
| | | | | | £89 | 6 | 7 |
| | | | | Balance carried forward ... ... ... | 21 | 16 | 11 |
| | £111 | 3 | 6 | | £111 | 3 | 6 |

Examined and found correct,

ALFRED JAMES HARRISON }
G. MUNRO SMITH } *Auditors.*

---

*November 14th, 1906.*

Mr. JAMES TAYLOR, President, in the Chair.

A proposal by Dr. Nixon for Mr. H. F. Mole, "That the May meetings of the Society be discontinued," was not carried.

The suggestion of Dr. Roxburgh that the next May meeting of the Society be held in Weston-super-Mare was approved.

Mr. W. ROGER WILLIAMS read a paper on **Tumours and Tubercle in Monkeys.** The maladies of monkeys were of great interest, because it was among the members of this order that the nearest approach in organisation to mankind was found. Notwithstanding the immense number of these creatures constantly under observation in the zoological collections of Europe, the author knew of only two modern instances of cancer in monkeys, and attempts to transmit experimentally human cancer to monkeys had invariably failed. It seemed clear that monkeys, like human savages, had very little proclivity to cancer. Their comparative immunity from non-malignant tumour was equally marked, for the author knew of only four modern instances of this kind. With regard to tubercle, it was an ancient belief that monkeys in captivity were very prone to it. Some years ago this was flatly contradicted by Bland-Sutton, but subsequent researches by G. H. J. Campbell, A. J. Harrison, Lydia Robinowstret and others had indubitably established the fact that monkeys in captivity were very prone to tubercle. It accorded with this that these creatures had proved to be very susceptible to experimental inoculation with both the human and bovine forms of tubercle. Thus in monkeys the same inverse relationship between cancer and tubercle was noticeable as in mankind.—Dr. SHINGLETON SMITH alluded to a case of tubercle in a chimpanzee. He had made a *post-mortem* examination of the animal sixteen years ago, had found that advanced tuberculous disease was the cause of death, and that tubercle bacilli were present in many organs.

Dr. J. A. NIXON read a paper on **Sclerodermia and Myositis,** showing three cases illustrating respectively "Sclerodermia *en plaques* with contraction of the tendon of the middle finger of one hand," "Sclerodermia affecting one arm associated with bone changes and tendon contraction," and "Generalised Myositis simulating rheumatoid Arthritis." The occurrence of inflammation of the muscles ending in contractions associated with such changes in the skin as are called "Sclerodermia" was regarded as being by no means very rare: records of at least fifteen such cases were quoted, several of which were verified by autopsy. In these cases no proof was found of any constant changes in the nerves or spinal cord. The probability of sclerodermia and the allied myositis being manifestations of some unknown inflammatory process was insisted upon, not as a novel suggestion but as a reversion to the ideas of the original observer of these conditions, Addison, whose monograph on "Keloid of Alibert and True Keloid" represents a more accurate and critical estimate of the disease than that of any subsequent observer until the researches of Thibierge were made known.—Dr. SWAIN asked if localised morphœa was the same disease in another form as diffuse sclerodermia.—Dr. CHARLES thought the cases shown bore some resemblance to cases of arsenical poisoning, and this suggested the possibility of a nerve lesion being the cause. He asked if thiosinamine had been used with any success in the treatment of sclerodermia.—Dr. EDGEWORTH said he had tried this drug for cirrhosis of the liver and mitral stenosis without effect. Some writers had reported good results from it, *e.g.* in cases of cicatricial stricture of the œsophagus.—Dr. MICHELL CLARKE alluded to the beneficial effect of the drug if administered hypodermically for Dupuytren's contraction. Dr. KENNETH WILLS had tried X-ray treatment for one case of diffuse sclerodermia without any beneficial result. He had not tried it for morphœa. Mr. ROGER WILLIAMS asked if there was any relationship between the myositis of sclerodermia and myositis ossificans.—The PRESIDENT remarked that he had examined one of the cases shown with the X-rays, and found marked atrophic changes in the phalangeal bones.

Dr. T. A. GREEN read a paper on **Heart Massage in cases of Sudden Syncope**, especially under anæsthetics. Certain physiological experiments were alluded to which had shown that rhythmical compression of the heart could restore the power of pulsation to the hearts of dogs and rabbits after these had been paralysed by asphyxia, chloroform and electric shocks. In these experiments artificial respiration had failed to restore cardiac pulsation. The speaker's first case was that of a boy aged 9, in whom, under chloroform anæsthesia, the heart beats and respiration had suddenly stopped. Artificial respiration and other restorative measures were first tried for about twenty-five minutes without success. The abdomen was therefore opened parallel to the left costal margin and the heart rhythmically compressed with one hand below the diaphragm and the other on the chest wall externally. After five minutes pulsation in the heart returned, and almost immediately spontaneous breathing was re-established. The patient did not recover consciousness. An hour later tonic spasms appeared in the limb and trunk muscles, and recurred at intervals for eight hours. The boy lived twenty hours. The author's second case was one of early diphtheria, in which syncope suddenly supervened in a child of three. The cardiac manipulations were begun about ten minutes after the cessation of pulsation, and were continued for three-quarters of an hour, as in the last case, through the diaphragm, without success. The diaphragm was then incised, and the heart grasped directly with three fingers in the pericardium, and after five minutes of rhythmical compression it began to beat regularly at the rate of forty-eight beats to the minute, and continued to beat for half an hour. Spontaneous breathing, however, could not be induced, and the efforts were at last reluctantly discontinued. Tracheotomy had been performed as one of the first steps for the restoration of the breathing in this case. The speaker then remarked that he had collected from literature thirty-nine cases in which heart massage had been applied for conditions such as those described above. Of these eight had been completely successful, in seven of which the manipulations had been carried out through an incision, and eight other cases had been partially successful. Three operative routes had been used—the trans-thoracic, abdominal trans-diaphragmatic, and the abdomino-sub-diaphragmatic. Of these methods the last was recommended as being quicker, and as having given the best results. Mr. Roger Williams mentioned the *post-mortem* evidence he had seen of rupture of the lungs and ecchymosis of the pericardium resulting from artificial respiration. This proved that the heart could be influenced by external manipulations.

J. Lacy Firth.
H. F. Mole, *Hon. Sec.*

---

### Obituary Notices.

